# Examining perceptions, social, and policy factors influencing hepatitis B birth-dose vaccine uptake: a PEN-3 cultural model mixed-methods study of healthcare workers in Nigeria

**DOI:** 10.3389/frhs.2026.1788089

**Published:** 2026-05-07

**Authors:** Olufunto A. Olusanya, Oluwakorede Adedeji, Caven Ngoe, David Oladele, Adesola Z. Musa, Maria Afadapa, Titi Gbaja-Biamila, Ifeoma Eugenia Idigbe, Peter Kalulu, Nkiruka Obodoechina, Temitope Ojo, Folahanmi Akinsolu, Abideen Salako, Joseph Ogbeh, Chiamaka Odinammadu, Ucheoma Nwaozuru, Kristi L. Foley, Agatha Wapmuk, Hong Xian, Jason J. Ong, Peyton Thompson, Oluwaseun Falade-Nwulia, Suzanne Day, Joseph D. Tucker, Oliver Chukwujekwu Ezechi, Juliet Iwelunmor

**Affiliations:** 1 St Louis John T Milliken Department of Medicine, Washington University, St. Louis, MO, United States; 2 Department of Clinical Pharmacy and Pharmacy Practice, University of Ilorin, Ilorin, Nigeria; 3 St Louis George Warren Brown School of Social Work, Washington University, St. Louis, MO, United States; 4 Nigerian Institute of Medical Research, Lagos, Nigeria; 5 Saint Louis University College for Public Health and Social Justice, St. Louis, MO, United States; 6 Department of Implementation Science, Wake Forest University School of Medicine, Winston-Salem, NC, United States; 7 Department of Clinical Research, Faculty of Infectious and Tropical Diseases, London School of Hygiene and Tropical Medicine, London, United Kingdom; 8 Division of Infectious Diseases, The University of North Carolina at Chapel Hill Institute for Global Health and Infectious Diseases, Chapel Hill, NC, United States; 9 Division of Infectious Diseases, The Johns Hopkins University School of Medicine, Baltimore, MD, United States

**Keywords:** healthcare workers, hepatitis Bvirus, newborns, PEN-3 cultural model, vaccines

## Abstract

**Introduction:**

The timely administration of the birth-dose hepatitis B (HepB-BD) vaccine is recommended by global and national guidelines to effectively prevent perinatal transmission. In high-disease-burden countries, such as Nigeria, the adoption of the HepB-BD vaccine remains limited. Emerging global policy discussions highlight the need to examine healthcare workers’ (HCWs’) perceptions of pediatric vaccination guidelines, the safety and effectiveness of the HepB-BD vaccine, and how family, social support, and community factors influence vaccine delivery.

**Methods:**

A convergent mixed-methods study of HCWs was conducted in Nigeria between July and August 2024. Using the PEN-3 Cultural Model as our framework, we concurrently administered quantitative surveys and conducted in-depth qualitative interviews and focus group discussions to assess knowledge, beliefs, and peer influences related to HepB-BD vaccinations. We also explored HCWs’ perceptions, enabling factors, and social influences that impact vaccine delivery. Data were analyzed using descriptive statistics and thematic analysis.

**Results:**

Most participants were women (87.8%), nurses (24.4%), and community extension workers (14.6%), with an average age of 32.5 years. Over half perceived HepB-BD vaccines as extremely safe (61%) and effective (63.4%). Participants strongly agreed that birth vaccination is recommended (80.5%), ethically appropriate (68.3%), and that its benefits outweigh side effects (63.4%). Most respondents reported professional autonomy in birth-dose decisions: 51.2% strongly disagreed that family opinions influenced their decisions, and 48.8% strongly disagreed that family/friends were concerned about their administering the vaccine. Qualitative themes included perceptions of barriers (poor knowledge, limited vaccine stock, misinformation), enablers (transportation and cold chain reliability), and nurturing factors (the role of training and collaboration with traditional birth attendants). Misconceptions included linking vaccines to sexually transmitted infections and concerns about fragile newborn immunity.

**Conclusion:**

HepB-BD vaccine delivery by HCWs is influenced by a complex interplay of multiple factors, including perceptions, structural enablers, and social relationships. As global policies continue to evolve, programs should adopt culturally responsive, context-specific strategies to address these multi-level influences and foster HCW trust, thereby ensuring the timely delivery of the HepB-BD vaccine.

## Introduction

Hepatitis B virus (HBV) infection, a major risk factor for liver cancer, remains a significant public health concern, causing more than a million deaths annually (1, 2). Africa experiences a disproportionate burden of chronic infections and HBV-related health problems and death (1). Nigeria is estimated to have the highest number of children affected by chronic HBV infection, primarily due to perinatal and early childhood transmission endemicity (3). Furthermore, the risk of developing chronic HBV infection is 90% when infection occurs during the perinatal period, compared to less than 5% with adult infection (3–5). This underscores the importance of timely administration of the hepatitis B birth dose (HepB-BD) vaccine, which reduces the risk of liver cancer (6). However, recent policy discussions among various actors (i.e., clinicians, policymakers, public health agencies, and professional societies) in high-income settings have considered shared clinical decision-making for specific maternal risk conditions (e.g., infection risk to newborn) to guide HepB-BD vaccination for newborns (7). This could further pose challenges to the implementation of the HepB-BD vaccine in low- and middle-income countries (LMICs), such as Nigeria, where the epidemiological context varies and vaccination delivery by healthcare workers (HCWs) is influenced by multiple factors, including limited knowledge of biomedical evidence, HCWs' misconceptions, vaccine shortages, staffing and health personnel issues, cost concerns, sociocultural norms, institutional trust, and vaccine policy interpretation (3).

The World Health Organization (WHO) recommends three doses of the HepB-BD vaccine, with the first dose administered to infants within 24 h of birth (3). Nigeria's immunization schedule allows the HepB-BD vaccine to be administered as part of routine immunization services to newborns within 24 h of birth and up to 2 weeks after, followed by doses at 6, 10, and 14 weeks as part of the pentavalent vaccine (8). Despite this national recommendation, a secondary analysis of data from the 2018 Nigeria Demographic and Health Survey, including 6,143 children aged 12–23 months, found that only approximately 53% had received the HepB-BD vaccine (9). While newborns delivered in public facilities are eligible for HepB-BD vaccination, those born in private facilities or by traditional birth attendants (TBAs) were less likely to receive the vaccine at all or in the recommended timeframe (9).

Provider-level and socio-cultural barriers to HepB-BD vaccine delivery include vaccine hesitancy, negative perceptions, misconceptions about HepB-BD, limited awareness of its safety and effectiveness, limited availability, infrastructure issues, poor transportation, food insecurity, and low health literacy level (8, 10, 11). At the system level, factors such as quality of service delivery, sustainable health financing, reliable vaccine procurement and supply, adequate storage and cold chain, and skilled personnel influence Nigeria's readiness for HepB-BD vaccinations (8–12).

In countries with a high disease burden, such as Nigeria, where healthcare workers (HCWs) may play an important role in linking evidence-based guidelines to community practice, it is essential to understand social and contextual factors to improve HepB-BD vaccine coverage. HCWs are the first point of contact for parents, act as trusted sources of information, and interpret vaccination guidelines to increase vaccine acceptance (13). Existing literature shows that knowledge, attitudes, and beliefs are important modifiable factors influencing HepB-BD vaccine administration among HCWs (3, 13). Moreover, individual trust in the safety and effectiveness of vaccines may influence perceptions and attitudes toward vaccinating infants against HBV, as well as perceptions of the benefits of the HepB-BD vaccine (14). Within our study context, the family is a key structural, social, and cultural factor that influences health and behavior. As an ecosystem, the family influences lifelong habits by modeling health behaviors or providing support to improve wellness and manage illness (15).

However, few studies have systematically explored these factors using theoretically grounded methods to understand how they influence the timely delivery of HepB-BD vaccines. Using the PEN-3 Cultural Model Framework, this study aimed to provide context-specific, exploratory insights into factors influencing healthcare workers' (HCWs) timely delivery of the HepB-BD vaccine to newborns by examining their perceptions of (1) HepB-BD vaccine safety and effectiveness, (2) the significance of family and social support, and (3) neighborhood and community factors influencing HepB-BD vaccine delivery.

## Methods

### Study design

Between July and August 2024, we conducted an exploratory, cross-sectional, mixed-methods study of HCWs in Lagos State, Nigeria. This study employed a mixed-methods design comprising two focus group discussions (FGDs; *n* = 21 participants), seven in-depth interviews (IDIs), and a quantitative survey (*n* = 41 respondents), allowing for triangulation of qualitative insights with concurrent quantitative findings. Two trained research assistants utilized the purposive sampling technique, leveraging health facility leadership and on-site engagement to recruit eligible community HCWs with relevant knowledge and experience in HepB-BD vaccine delivery. To improve the transferability of findings and increase diverse perspectives among HCWs, the eligibility criteria included HCWs from various professional roles and backgrounds (i.e., physicians, nurses, midwives, community extension workers, and labor and delivery personnel) who provide, coordinate, or oversee maternal, newborn, or HepB-BD vaccine delivery services. HCWs who were not engaged in perinatal or newborn care were excluded. We also recruited HCWs employed in multiple healthcare settings to increase variations in perspectives. The questionnaires were pilot-tested among a separate set of 20 HCWs to assess clarity, relevance, and cultural appropriateness of the survey instrument before the study was conducted.

### Study setting

The study was conducted in Lagos State, Nigeria, with an estimated population of about 22 million. Lagos is divided into 20 local government areas (LGAs), 16 urban and 4 rural. The state has 2,333 health facilities: 1,574 primary, 756 secondary, and 3 tertiary. Among these, 458 are public and 1,875 are private (16). The ratios of residents to general medical doctors and midwives are roughly 5,014 to 1 and 5,117 to 1, respectively, both significantly higher than the WHO recommendation (16). Lagos had the highest immunization coverage in the southwestern region of Nigeria at 66%; however, some LGAs reported coverage below 60% (17). Lagos State's estimates of HepB birth dose coverage are limited in the published literature; however, national data indicate that approximately 53% of Nigerian newborns receive the birth dose vaccine (9).

### Theoretical framework

The PEN-3 cultural model, developed by Airhihenbuwa (1989), highlights the centrality of culture, norms, social relationships, and enabling structures in shaping health behaviors and emphasizes three interrelated domains: “Cultural Identity,” “Relationships and Expectations,” and “Cultural Empowerment” (18). For our study, the “Relationships and Expectations” and “Cultural Identity” domains were employed to examine HCWs' perceptions, enablers, and nurturers, as well as the influence of individual beliefs, social support, institutional structures, and community expectations. These domains are well-suited to our study because decisions regarding the administration and delivery of HepB-BD vaccines are often influenced by the complex interplay of perceptions, knowledge, and capacity, institutional infrastructure and support, and community norms (19). Emerging discussions on HepB-BD vaccine policy highlight that vaccine guidelines can be interpreted and implemented differently across health system contexts, emphasizing the importance of applying culturally grounded frameworks that consider HCWs and the social settings in which they practice (7, 20).

### Measures assessed

Using the PEN-3 cultural model, the following measures were assessed quantitatively and qualitatively.

#### Vaccine perceptions

Examples of questions quantitatively assessing HCWs' vaccine perceptions were “Babies at birth should get the HepB-BD vaccine to avoid liver cancers?” “To what extent do you think the HepB-BD vaccine is safe for newborn babies?” and “The benefits of the HepB-BD vaccine in preventing liver cancer are better than any vaccine side effects.” Moreover, perceptions of the acceptability, burden, and ethics of vaccine administration were also assessed: “How acceptable do you think it is to provide HepB-BD vaccines to newborn babies?” and “I think there are moral or ethical consequences with giving HepB-BD vaccines to newborns.” Other questions assessed perceived benefits, effort, time, and resource burden. Qualitatively, one of the questions that assessed perception was “What are your thoughts on the safety of the HepB-BD vaccine among babies?”

#### Perceived neighborhood and community-level influences on HepB-BD uptake

Community- and neighborhood-influences were evaluated to understand HCWs' perspectives on the broader social and structural environment in which they live and how these affect their perceptions, decision-making, and ability to deliver timely HepB birth dose services. HCWs' perceptions of neighborhood and community factors were examined through questions like: “How have things changed for you or your household regarding: safe housing, access to nutritious food, medical care access, transportation, education, and job opportunities?” Qualitatively, one question asked was “How easily can you access HepB-BD vaccines in your clinic?”

#### Role of family and social support

HCWs' perceptions of their family and peer support for administering the HepB-BD vaccines were assessed: “My family will support me as I administer the birth-dose HBV vaccines,” “The opinion of my spouse and family affect my decision to administer the birth-dose HBV vaccines to babies,” and “My family or friends are concerned about me administering the birth-dose HBV vaccine to babies.”

### Data collection instrument and ethical considerations

The quantitative study used a self-administered structured questionnaire, delivered in person and online in English, with four sections: (1) sociodemographic, (2) perceptions of vaccine benefits, safety, ethics, and effectiveness using Likert-scale multiple-choice questions, (3) community and neighborhood influences, and (4) family and peer support. Additionally, the qualitative data collection explored HCWs' perceptions, awareness, and understanding of pregnant and postpartum women's needs, public health education, community support, vaccine benefits and risks, confidence, social determinants, trusted information sources, and misinformation.

The Health Research Ethics Committee of the Nigerian Institute of Medical Research approved the study. Informed consent was obtained from all participants while ensuring voluntary participation.

### Statistical analysis

Participants' demographic characteristics were reported as descriptive summary statistics. Measures of central tendency, such as mean and median, were performed. Spearman's rank correlation coefficient was computed using STATA, version 18 (StataCorp LLC, College Station, TX, USA), to evaluate the correlation between the two variables. A *p*-value of less than 0.05 was considered statistically significant.

Guided by the PEN-3 cultural model, we conducted a six-step thematic analysis using a deductive approach to systematically examine participants' responses to the qualitative open-ended questions. First, we reviewed the qualitative responses and then generated initial codes by systematically highlighting features of the data relevant to the PEN-3 domains. Thirdly, these codes were collated into potential themes and sub-themes. In the fourth step, we established and labeled themes to ensure clarity and alignment with the “Relationships and Expectations” and “Cultural Identity” domains of the PEN-3 framework. We then selected impactful quotes that effectively illustrated each theme. Finally, we organized themes and sub-themes within their respective PEN-3 domains. Participants were recruited for the qualitative study until no new themes emerged, in accordance with qualitative research standards.

## Results

### Quantitative study

Most were females (87.8%), married (48.8%), and Christians (85.4%). The mean age of the respondents was 32.5 (SD = 11.5). All respondents had completed tertiary education. The respondents included nurses (24.4%), community health extension workers (CHEWs) (13.0%), individuals in professional/technical/managerial roles (14.6%), and medical assistants (9.8%) (see Table 1).

**Table 1:** Characteristics of study participants (*N* = 41)

Participants’ characteristics	Summary [*n* (%)]
Gender	
Female	36 (87.8%)
Male	5 (12.2%)
Age at last birthday (years)	
Median (Min, Max)	28.0 (17.0, 57.0)
Marital status	
Married	20 (48.8%)
Single	20 (48.8%)
Religion	
Christian	35 (85.4%)
Muslim	6 (14.6%)
Highest level of education	
Tertiary	41 (100%)
Ethnic group	
Hausa	2 (4.9%)
Igbo	7 (17.1%)
Yoruba	28 (68.3%)
Other	4 (9.8%)
State of residence	
Lagos	40 (97.6%)
Geographic location where you live	
Rural	2 (4.9%)
Urban	39 (95.1%)
Employment status	
Full-time	30 (73.2%)
Part-time	7 (17.1%)
Unemployed	2 (4.9%)
Other	2 (4.9%)
Occupation	
Nurse	10 (24.4%)
Community Health Extension Workers	6 (14.6%)
Professional/technical/managerial	5 (12.2%)
Medical assistant	4 (9.8%)
Student	4 (9.8%)
Pharmacist	2 (4.9%)
Health Information Management Officer	2 (4.9%)
Doctor	2 (4.9%)
Midwives	1 (2.4%)
Other	5 (12.2%)
Level of health facility of employment	
Primary	29 (70.7%)
Secondary	1 (2.4%)
Tertiary	10 (24.4%)

#### Perceptions of vaccine benefits, safety, and effectiveness

**Figure 1:**
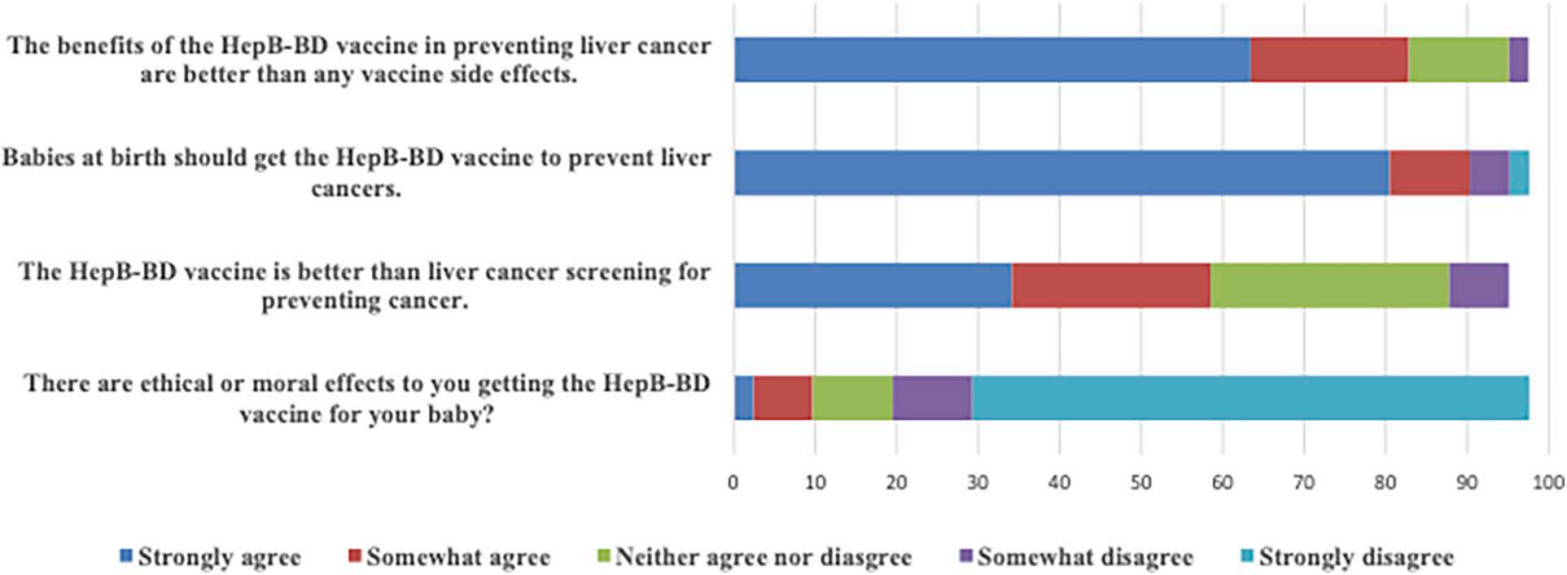
Perceptions of vaccine benefits and ethics.

More than half of the participants indicated that they “strongly agreed” with the following statements: “Babies at birth should get the HepB-BD vaccine to avoid liver cancers” (80.5%), “The benefits of the HepB-BD vaccine in preventing liver cancer are better than any vaccine side effects” (63.4%). A smaller proportion strongly agreed that “The HepB-BD vaccine is better than liver cancer screening for preventing cancer” (34.1%), while very few participants strongly agreed that “There are ethical or moral concerns about giving my baby the HepB-BD vaccine” (2.4%). See Figure 1. About 61.0% and 63.4% of respondents perceived HepB-BD vaccines as “extremely safe” and “extremely effective,” respectively, for newborns. Most respondents expressed that administering the HepB-BD vaccine required “none at all” effort (60.9%), resources (50.0%), or time (47.8%).

Perceived benefits of the HepB-BD vaccine in preventing liver cancer were weakly but significantly correlated with perceived vaccine safety among community HCWs (*r* = 0.38; *p* = 0.018). Perceived safety was perfectly correlated with vaccine confidence (*r* = 1.00; *p* < 0.001), indicating that HCWs who view the vaccine as safe are more likely to support its administration at birth.

#### Neighborhood and community factors as enablers of timely HepB-BD vaccine delivery

When asked about how have things have changed for them or them household overtime, HCWs responded that the following were “somewhat/much better”: transportation (41.5%), job opportunities (53.7%), safe neighborhoods (56.1%), HCW shortage (26.8%), gender equality (39.0%), and government support (41.5%). See Table 2.

**Table 2:** Perceived neighborhood and community factors that could influence hepB-BD vaccine delivery as reported by healthcare workers.

Characteristics	Somewhat/much worse	About the same	Somewhat/much better
Safe housing	3 (7.3%)	11 (26.8%)	26 (63.4%)
Emotional health	4 (9.8%)	10 (24.4%)	26 (63.4%)
Access to nutritious food	6 (14.6%)	6 (14.6%)	28 (68.3%)
Access to medical care	4 (9.8%)	11 (26.8%)	25 (61.0%)
Transportation	12 (29.3%)	11 (26.8%)	17 (41.5%)
Education	3 (7.3%)	12 (29.3%)	25 (61.0%)
Job Opportunities	7 (17.1%)	11 (26.8%)	22 (53.7%)
Security and safe neighbourhoods	9 (22.0%)	8 (19.5%)	23 (56.1%)
Education and literacy skills	1 (2.4%)	13 (31.7%)	26 (63.4%)
Healthcare worker shortages	12 (29.3%)	17 (41.5%)	11 (26.8%)
Gender equality	5 (12.2%)	19 (46.3%)	16 (39.0%)
Government support	9 (22.0%)	14 (34.1%)	17 (41.5%)

#### Family and peer support as nurturers of timely HepB-BD vaccine delivery

**Figure 2:**
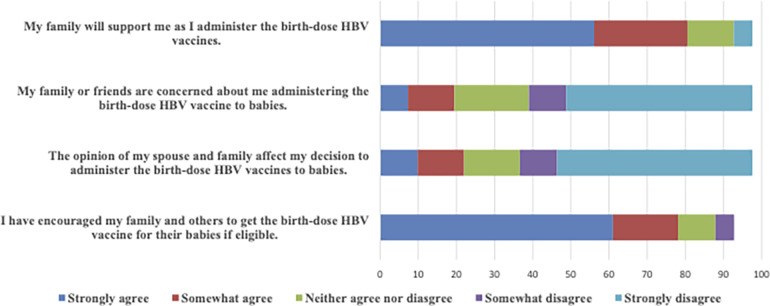
Family and peer support in administering the HepB-BD vaccine.

HCWs' responses regarding family and peer support, opinions, and concerns varied when assessed regarding vaccine delivery. While 80.5% “strongly/somewhat agreed” that their family would support their HepB-BD vaccine delivery, 19.5% “strongly/somewhat agreed” that their family or friends expressed concern about them administering the HepB-BD vaccine to newborns. Overall, 22.0% of respondents “strongly/somewhat agreed” that their spouses or family's opinions influenced their decision to administer the birth-dose vaccine. In contrast, 78.1% “strongly/somewhat agreed” that they had encouraged their family and others to receive the HepB-BD vaccine. See Figure 2.

There was no statistically significant relationship between family support and decision to administer the HepB-BD vaccine among HCWs (*r* = −0.24; *p* = 0.151), indicating that family support, whether present or absent, did not affect HepB-BD vaccine delivery. Also, concerns from family or friends about HCWs' vaccine delivery were not statistically significantly associated with perceived vaccine safety (*r* = −0.23; *p* = 0.182). The assumptions for correlation analyses, including normality and linearity, were assessed and satisfied before conducting the tests. Most HCWs preferred to obtain their health information online. Training or workshops were also essential sources of health information.

### Qualitative study

Participants from the two FGDs and 7 IDIs were HCWs with tertiary education, mostly residing in Lagos. The mean age was 36.25. The qualitative analysis revealed three key themes within the Relationship and Expectation: (1) perceptions of HepB-BD vaccination for newborns, (2) enabling factors influencing HepB-BD vaccination, and (3) nurturing factors shaping decisions and actions on HepB-BD vaccination. See Table 3.

**Table 3:** Other relevant quotes categorized by two domains from the PEN-3 cultural model among HCWs.

Relationship and expectations domain	Cultural identity domain	Theme	Illustrative quotes
Perceptions	Positive	Knowledge	“It is a viral infection… a chronic disease that attacks the liver and can lead to cirrhosis and cancer.”
	Positive	Knowledge	“It is to prevent mother-to-child transmission and early exposure.”
	Positive	Perceived benefit and effectiveness	“Vaccinated children stand a higher chance of not being affected.”
	Positive	Perceived vaccine effectiveness	“It's not only better, it is cheaper and the most important thing to do. Prevention is better than cure.”
	Positive	Perceived vaccine effectiveness	“Getting hepatitis B dose for a child within 24 h prevents liver problems like 98%, and that is huge.”
	Positive	Perceived vaccine safety	“Very, very safe. I’ve given a lot of babies hepatitis B vaccine and I’ve never had any complications.”
	Existential	Perceived risk	“Even for babies that will react within the first 24 h, you can give them paracetamol and they will be fine.”
	Existential	Perceived risk	“There is nothing in this world without risk, even water.”
	Existential	Perceived risk	“Reaction at the site, pain, fever… all this can be handled.”
	Negative	Limited knowledge	“Some mothers don’t have adequate knowledge… when you try to convince them, they react negatively.”
	Negative	Beliefs	“It's an injection given to women… to prevent cervical cancer.”
	Negative	Beliefs	“People who go about giving vaccines are not proper health workers.”
Enablers	Positive	Training and self-efficacy	“We are trained… we check VVM, expiry date, batch number.”
	Positive	Training and self-efficacy	“The training has prepared me. The experience has equipped me.”
	Positive	Vaccine availability	“Very, very easily accessible. At a snap of the finger, you get your hepatitis B vaccine.”
	Positive	Timeliness of HepB-BD vaccine delivery	“If it's still within 48 h, we still administer.”
	Positive	Vaccine cost	“It is free of charge.”
	Negative	Structural barriers	Power supply issues, cold-chain gaps, distance, time wastage, and stock-outs.
	Negative	Vaccine shortage	“More people are aware but fewer vaccines are available.”
	Negative	Health provider shortage	“Health workers are leaving the country… short staff affects dissemination.”
Nurturers	Positive	Health provider education	“My mouth is for education; I keep talking.”
	Positive	Community support	“Community is like a family-based unit. They know who just gave birth.”
	Negative	Limited community engagement	“When people are not carried along, they resist.”
	Negative	Health provider distrust	“Perhaps they may not trust the expertise of the healthcare worker.”
	Negative	Cultural resistance	“They don’t like taking immunisation… based on cultural beliefs.”

#### Perception towards hepB-BD vaccinations of newborns

This theme explored participants' perceptions of HBV, vaccination, and liver cancer. Hepatitis was widely recognized as a significant risk factor for liver cancer, and participants identified preventive measures such as HBV vaccination, reducing alcohol use, and cautious drug use. See Table 3. Most knowledgeable about HBV as a liver disease that can lead to cirrhosis and cancer, and demonstrated awareness of its transmission routes and severity.

“Hepatitis B is a virus that attacks the liver… if not treated it can lead to liver cirrhosis and cancer.”

Participants generally viewed vaccination as essential for immunity and effective in protecting against HBV infection, with some emphasizing its role in preventing liver cancer.

“It is a proactive measure… so that the baby will not be exposed at the early stage of life.”

“Prevention is better than cure… giving it at birth is better than screening later.”

“It has been verified that it can reduce it up to 99% if given within 24 h.”

However, some participants inquired about how to better understand the vaccine and reported low community awareness and vaccine refusal due to beliefs and norms.

“Hepatitis B is usually sexually contacted… I think it is not necessary at birth.”

“Some mothers don’t have adequate knowledge… when you try to convince them, they react negatively.”

“Some parents refuse due to past complications or beliefs.”

Opinions varied regarding the optimal timing for vaccine administration. While most agreed on administering it within 24 h of birth, a few advocated a delay, with one participant arguing that if a child is not exposed, vaccination can be scheduled for later in life.

“… it is not necessary at birth, except when the child is exposed to injectables and all that. I think that it is something that they can still take, maybe four months or six months, instead of at birth.”

#### Enabling factors influencing HepB-BD vaccine delivery

This theme described the vaccination process and the health system's readiness to administer HepB-BD vaccines. Participants emphasized the importance of reliable access to vaccines at prenatal clinics. They identified enablers such as training, outreach support from community mobilizers, and the availability of information in socio-culturally tailored languages.

“I have been trained… I know what I am doing.”

“ Hepatitis B has been passed down to health workers through health training, workshops, and it has been mandated that every child that has been delivered in the facility must take the Hepatitis vaccine….”

“Posters, flyers, and megaphones were provided to create awareness.”

“We use jingles, leaflets in local language, and health talks during antenatal clinics.”

Participants emphasized that infrastructure, such as reliable power, cold-chain capacity, transport, and adequate funding, posed barriers to effective vaccine delivery.

“You don’t have light to store the vaccine. That can be a barrier.”

“Some places are very far from the healthcare center.”

One participant also requested support for traditional birth attendants.

“I think it’s also crucial to provide resources to traditional birth attendants either to get the vaccine and also to have the good chain system….”

“Community leaders introduce us to traditional birth attendants and mothers who just gave birth.”

Facilitators reported for HepB-BD vaccine uptake among mothers of newborns included integration into routine immunization workflows, vaccine cost, vaccination reminders, incentives, and effective communication.

“Any child delivered here receives zero-day vaccination immediately.”

“The immunoglobulin costs about ₦60,000 (i.e., $41); government support is needed.”

#### Nurturing factors shaping decisions and actions on HepB-BD vaccine delivery

Nurturing factors were perceived as social relationships and interpersonal influences that foster trust and support for timely HepB-BD vaccine delivery. Implementation strategies such as community engagement, parental/caregiver education, antenatal counseling, outreach efforts, and partnerships with community leaders and traditional birth attendants (TBAs) were considered effective for enhancing vaccine delivery.

“From antenatal to postnatal, mothers are taught about the vaccine.”

“We first go to the community head before organizing outreach.”

Nevertheless, community support varied, and in some areas, vaccine hesitancy driven by cultural beliefs and misinformation was demonstrated.

“Support from community leaders has been poor.”

## Discussion

This study utilizes the PEN-3 Cultural Model to examine context-specific, exploratory insights into factors influencing HCWs' perceptions of (1) the vaccine's safety and effectiveness, (2) family and social support, and (3) community factors influencing timely HepB-BD vaccine delivery. Quantitatively, more than half of the respondents viewed the vaccine as safe or effective and believed that its benefits outweighed its risks. More than half reported family support for their HepB-BD vaccine delivery. Qualitative findings identified three key themes: perceptions of the HepB-BD vaccine, enabling factors for timely vaccine delivery, and nurturing influences on vaccination decisions.

Without intervention, babies who are exposed through mother-to-child transmission face a lifetime risk of developing cirrhosis or hepatocellular carcinoma. In general, many HCWs held a positive perception of the HepB-BD vaccines; over half of our participants perceived HepB-BD vaccines to be safe and effective. Our findings are consistent with similar research among healthcare professionals in Turkey and Ethiopia, in which more than half of the participants acknowledged the benefits of the HepB-BD vaccine (10, 11). A study in Nigeria also demonstrates that HCWs recognized the importance of the HepB-BD vaccine (3). In addition, Moturi et al. reported that many HCWs interviewed in Botswana and São Tomé and Príncipe were knowledgeable about the HepB-BD vaccine and its effectiveness when administered within 24 h (3).

Despite the proven effectiveness of timely HepB-BD vaccination, negative perceptions among HCWs can limit the impact of vaccination programs. Overall, less than 5% of HCWs considered the HepB-BD vaccine unsafe or ineffective, and 2.4% believed its risks outweighed its benefits. Additionally, 2.4% expressed ethical or moral concerns about vaccinating infants. These negative perceptions among only a few of the HCWs may stem from limited knowledge, concerns about vaccine side effects and effectiveness, and misconceptions. A study of 80 Nigerian health facilities found that HCWs falsely believed misinformation, leading to lower vaccination rates among preterm and low-birth-weight infants (21). Additionally, a study in Vietnam found that many providers feared adverse events following vaccinations, including the HepB-BD vaccine, due to concerns about parental blame and potential legal repercussions (22), while vaccine safety concerns were commonly expressed among HCWs (23–25). In Cameroon and Nigeria, studies indicate that vaccine safety concerns and fears about the HepB-BD vaccine's side effects were prevalent among HCWs (12, 24). Providing HCWs with training on the benefits of timely vaccination, as well as the safety and effectiveness of HepB-BD vaccines, could reduce negative perceptions, improve vaccine acceptance, and increase self-efficacy in administering vaccines.

Family and peer support reflect social relationships and interpersonal influences that serve as nurturing factors that could reinforce timely HepB-BD vaccine delivery. Most participants (56.1%) believed their families would support their decision to deliver the HepB-BD vaccine. Only a few respondents (9.8%) strongly agreed that their spouses' and families' opinions influenced their decision to administer the birth-dose vaccine. Additionally, some HCWs expressed willingness to encourage their families and others to get the birth-dose vaccine for their babies if they are eligible. Similar patterns of family influence on HCW vaccination decisions were observed in a 2022 barrier analysis of COVID-19 vaccinations among HCWs in Ilorin, Kwara State. The study found that 97% of participants were willing to be vaccinated if they received support from family members or close friends (26). Although few studies have examined the impact of family and peer support on vaccine delivery among HCWs, future research should investigate the role of this positive motivator in improving timely birth dose vaccination rates. Moreover, a review of studies focused on HCWs demonstrated that when vaccine hesitancy was observed among family, friends, or peers, HCWs were more likely to be hesitant about vaccines (27). Bradshaw et al. highlighted the importance of collaboration and family education in increasing HepB-BD vaccination rates (28).

Neighborhood and community factors refer to systems and structures that can hinder or facilitate HCWs' vaccine delivery. The neighborhood encompasses various elements, including infrastructure, socioeconomic conditions, cultural, and political factors. At the time of our study, about half of HCWs perceived better job opportunities (53.7%) and neighborhood safety (56.1%), while fewer reported improvements in transportation (41.5%) and government support (41.5%). Only 26.8% perceived a reduction in healthcare worker shortages (26.8%). These enabling factors influence the health system environment and are likely to facilitate the timely delivery of HepB-BD vaccine.

Previous studies have reported a link between birth date and the likelihood of receiving the HepB-BD vaccine (4). In their study, Bada et al. reported that infants born on Mondays through Thursdays were more likely to receive the timely dose of the HepB-BD vaccine compared to those born on Fridays (4). The practice of HCWs limiting vaccination services to specific days of the week, rather than offering daily vaccination, has been frequently cited in studies as a key contributor to delayed vaccine administration, including for the HepB-BD vaccine (9, 29). This scheduling approach can create barriers to timely immunization, particularly when a child's birth does not coincide with designated vaccination days. Addressing this issue requires coordinated efforts between vaccination teams and maternity care providers, including midwives, nurses, and doctors involved in labor and delivery. Moreover, further planning and logistical support are essential for newborns delivered outside health facilities, such as during home births—a common occurrence in many low-resource settings. Strategies may include deploying community health workers or birth attendants to promptly notify health facilities after a home delivery, and organizing reliable, rapid transport mechanisms to ensure newborns reach vaccination sites within the recommended window period (3).

### Implications for HepB-BD vaccine in a global policy landscape

Ongoing discussions in high-income countries have focused on the roles of HCWs' discretion and shared decision-making in the delivery of the HepB-BD vaccine to newborns. Although epidemiologic and health system contexts in our study setting differ from those in other settings, ongoing discussions and findings emphasize that HCWs are essential to translating policy into practice. This study indicates that multilevel interventions that address negative perceptions, enablers, and nurturing factors influencing HCWs' timely delivery of the HepB-BD vaccine ought to inform future implementation strategies and research. In Nigeria, where the disease burden is high, ambiguity in vaccination guidelines and variability in HCWs' perceptions of birth-dose timing, vaccine safety, and efficacy could have unintended consequences. Interventions that address HCWs' awareness of vaccine safety, benefits, and effectiveness may be inadequate without integration with broader societal and political contexts, institutional constraints, and community norms.

In the context of our study, our exploratory findings emphasize the need for clear, unambiguous, evidence-based vaccination guidelines to reduce misconceptions and promote collective vaccine acceptance. Moreover, adopting culturally appropriate implementation strategies that train HCWs, engage their families and trusted community members, and address structural barriers may be crucial to enhancing HepB-BD vaccine delivery among newborns in the local setting.

### Limitations

While our study provided insights into HCWs' perceptions, enablers, and social influences on HepB-BD vaccine uptake, several limitations must be acknowledged. The small, purposively sampled study conducted in one Nigerian state may introduce sampling bias and limit the generalizability of findings to other regions and populations (e.g., mothers and caregivers). The self-reported data collection could also lead to social desirability bias. Moreover, since some survey items assessed similar constructs, the resulting measures exhibited strong correlations, which likely explains the perfect correlation observed. Despite these limitations, this study offers preliminary insights into HCWs' perceptions, enablers, and social influences on HepB-BD vaccine delivery.

## Conclusions

High-disease-burden countries such as Nigeria should reinforce the critical importance of timely universal HepB-BD vaccine delivery. Exploring HCWs' lived experiences, sociocultural factors, and health system contexts using frameworks such as PEN-3 can facilitate more context-specific implementation strategies that increase HepB-BD vaccine delivery and reduce missed opportunities for vaccinations at birth.

## Data Availability

The original contributions presented in the study are included in the article/Supplementary Material, further inquiries can be directed to the corresponding authors.
